# A Semi-Supervised Multi-Region Segmentation Framework of Bladder Wall and Tumor with Wall-Enhanced Self-Supervised Pre-Training

**DOI:** 10.3390/bioengineering11121225

**Published:** 2024-12-04

**Authors:** Jie Wei, Yao Zheng, Dong Huang, Yang Liu, Xiaopan Xu, Hongbing Lu

**Affiliations:** 1 School of Biomedical Engineering, Air Force Medical University, No. 169 Changle West Road, Xi’an 710032, China; jieweiwtt@fmmu.edu.cn (J.W.); zhengyao0202@fmmu.edu.cn (Y.Z.); huangdong1007785@outlook.com (D.H.); yliu@fmmu.edu.cn (Y.L.); 2 Shaanxi Provincial Key Laboratory of Bioelectromagnetic Detection and Intelligent Perception, No. 169 Changle West Road, Xi’an 710032, China

**Keywords:** bladder cancer, magnetic resonance image, self-supervised learning, multi-region segmentation, semi-supervised learning

## Abstract

Bladder cancer is a prevalent and highly recurrent malignancy within the urinary tract. The accurate segmentation of the bladder wall and tumor in magnetic resonance imaging (MRI) is a crucial step in distinguishing between non-muscle-invasive and muscle-invasive types of bladder cancer, which plays a pivotal role in guiding clinical treatment decisions and influencing postoperative quality of life. The performance of data-driven methods is highly dependent on the quality of the annotations and datasets, however the amount of high-quality annotated data is very limited given the difficulty of professional radiologists to distinguish the mixed regions between the bladder wall and the tumor. The performance of the data-driven approach is highly dependent on the quality of the annotation and datasets, Therefore, in order to alleviate these problems and take full advantage of the potential of limited annotated and unlabeled data, we designed a semi-supervised multi-region framework for bladder wall and tumor segmentation. Our framework incorporates wall-enhanced self-supervised pre-training, designed to enhance discrimination of the bladder wall, and a semi-supervised segmentation network that utilizes both limited high-quality annotated data and unlabeled data. Contrast consistency and reconstruction observation losses are introduced to constrain the model to enhance the bladder walls, and adaptive learning rate and post-processing techniques are implemented to further improve segmentation performance. Extensive experimental validation demonstrated that our proposed method achieves promising results in the segmentation of both the bladder wall and the tumor. The average Dice Similarity Coefficients (DSCs) of the proposed method for the bladder wall and tumor were 0.8351 and 0.9175, respectively. Visualization results indicated that our method can effectively reduce excessive segmentation artifacts outside the bladder, and improve the clinical significance of the segmentation results.

## 1. Introduction

Bladder cancer is a prevalent and highly recurrent malignant tumor of the urinary tract, ranking among the top ten cancers globally in terms of both incidence and mortality [1]. According to the latest estimates from the International Agency for Research on Cancer, there were approximately sixty thousand new cases of bladder cancer and twenty thousand related deaths globally in 2022 [2]. According to the tumor T staging, bladder cancer can be divided into two distinct categories: non-muscle-invasive bladder cancer (NMIBC, stages T1 or lower) and muscle-invasive bladder cancer (MIBC, stages T2 or higher), mainly depending on whether the tumor infiltrates the muscle or not [3]. Clinically, different T staging statuses necessitate distinct treatment strategies and prognostic management [4,5,6]. For example, the typical treatment for NMIBC is a transurethral resection or adjuvant therapy combined with photodynamic chemotherapy [7], whereas MIBC usually requires a radical cystectomy or bladder preservation treatments [8]. Accurate delineation of the bladder wall and tumor boundary is one of the pivotal prerequisites for physicians to determine whether the tumor infiltrates the muscle and further confirm the tumor stage. Therefore, accurate segmentation of the bladder wall and tumor is crucial for determining the stage of bladder cancer, guiding clinical treatment decisions and influencing postoperative quality of life.

Currently, the staging diagnosis of bladder cancer primarily relies on biopsy results obtained via optical cystoscopy [9]. However, optical cystoscopy is an invasive examination, making it susceptible to causing secondary injuries to patients. Additionally, it only captures the local characteristics of the lesion and cannot observe the invasion of the tumor on the bladder wall. Consequently, it is challenging to provide a comprehensive assessment of a tumor using this technique, often leading to underestimations or misjudgments of the tumor stage [10,11]. Preoperative magnetic resonance imaging (MRI), an important clinical non-invasive examination, offers a comprehensive evaluation of the tumor and its surrounding tissues, playing an essential role in the precise diagnosis and treatment of bladder cancer. While T2-weighted imaging provides a clear visualization of organ structures, less experienced radiologists often face difficulties in differentiating the bladder wall and tumor due to similar intensity distributions or blurred boundaries. Furthermore, manual segmentation is a time-consuming process that relies heavily on the experience of the operator, which can introduce variability. Therefore, the development of automated algorithms to assist physicians in accurately segmenting the bladder wall and tumor has attracted much research attention.

Over the past decades, various approaches for bladder wall and tumor segmentation have been proposed, and can be primarily categorized into model-driven and data-driven methods. Model-driven methods tend to use classical machine learning techniques, deformable models [12,13,14,15], clustering [16], or classifiers trained by handcrafted features [17]. The objective functions of these methods are initially formulated based on prior knowledge and then minimized to achieve the desired objective segmentation. Duan et al. [18,19] presented a volume feature and adaptive window setting method following the coupled level sets to achieve bladder cancer segmentation which combines regional adaptive clustering with coupled level sets to iteratively segment inner and outer walls. Xiao et al. [16] modified the introduction of the spatial fuzzy c-means algorithm with the spatial domain information of the inner and outer bladder walls to segment the cancer regions. Li et al. [20] first combined an Expectation and Maximization algorithm with a Markov Random Field to segment the bladder wall, then used region growing to obtain the region of the bladder lumen. Liu et al. [17] extracted the voxel features of the bladder wall and cancer to train a support vector machine which is used for segmenting the bladder cancer from the bladder wall obtained via coupled level sets. However, these model-driven algorithms rely on handcrafted features and local optimization, and have high sensitivity to initialization; hence, they provide a limited representation of complicated bladder wall and tumor mixed regions. Moreover, most of these methods are limited to segmentation of the bladder wall alone and lack the capability to simultaneously segment both the wall and tumor, which restricts their clinical applications.

Deep learning models as representative models for data-driven methods have been widely employed in bladder segmentation [21,22,23,24,25,26]. Recently, deep convolutional neural networks (DCNNs) have been successfully applied to many medical image segmentation tasks, and this has driven the adoption of DCNNs for bladder tumor segmentation, particularly U-Net and its variants [27,28,29,30]. Dolz et al. [22] introduced a multi-region bladder segmentation network leveraging progressively dilated convolutions to broaden the receptive field, achieving remarkable results in bladder wall segmentation. Liu et al. [31] advanced this field by integrating pyramid networks with U-Net, enabling multi-scale segmentation of tumors and bladder walls to be performed in an end-to-end manner. Ma et al. [32] conducted a comparative analysis between 2D and 3D U-Nets for bladder segmentation, highlighting their respective strengths. Dong et al. [33] incorporated attention mechanisms to integrate bladder shape priors into the segmentation model, thereby introducing the content and shape attention network (CSAN), which enhances segmentation accuracy by emphasizing pertinent image features associated with tumor segmentation. Data-driven methods can learn image representations from data in a unified framework, thereby avoiding the cumbersome processes of handcrafted feature extraction and classifier design. However, as they are supervised solutions, their performance is highly dependent on the quality of the annotation and dataset.

**Figure 1:**
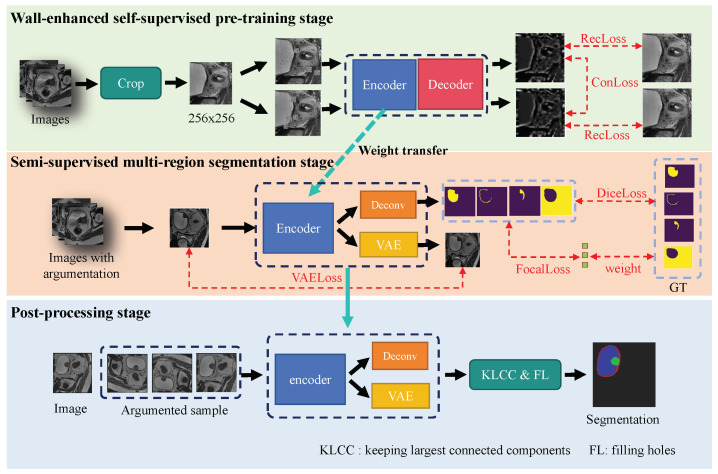
Overview of the proposed semi-supervised multi-region framework for bladder wall and tumor segmentation with wall-enhanced self-supervised pre-training. The method has three stages: the wall-enhanced self-supervised stage for pre-training, semi-supervised training stage for bladder wall and bladder segmentation, and a post-processing stage for segmentation result augmentation and correction.

Due to the difficulty in distinguishing mixed regions between the bladder wall and tumors, even professional radiologists do not necessarily provide completely accurate labels for every image. Thus, datasets for bladder cancer segmentation are usually mixed, with high-quality and low-quality labels, and have limited size. Therefore, in order to mitigate these issues and harness the full potential of limited labeled data and unlabeled data, we designed a semi-supervised multi-region framework for bladder wall and tumor segmentation with wall-enhanced self-supervised pre-training, shown in Figure 1. The main contributions of the paper can be summarized as follows:

- We designed a wall-enhanced self-supervised pre-training method using contrast consistency and reconstruction observation losses to improve the network’s ability to distinguish bladder walls. The visualization results show that the bladder walls could be more easily differentiated, which benefited the subsequent segmentation of the mixed regions of tumors and bladder walls by our segmentation network.
- We introduced a semi-supervised multi-region framework for bladder wall and tumor segmentation with a variations auto-encoder (VAE) branch, which transfers the weights from the pre-training stage and is trained with high-quality annotations and unlabeled data. We applied an adaptive learning rate strategy and Dice focal loss to further increase the segmentation accuracy for bladder walls and tumors.
- We designed a multi-view augmentation testing strategy that involves post-processing such as keeping the largest connected component and filling holes to further improve and correct segmentation results.

## 2. Materials and Methods

The proposed method encompasses three distinct stages depicted in Figure 1. Firstly, in the wall-enhanced self-supervised pre-training stage, we introduce a novel approach centered on self-supervision aimed at image reconstruction, which introduces contrast consistency and reconstruction observation losses to enhance the boundary of the reconstructed bladder wall. Then, in the semi-supervised multi-region segmentation stage, we transfer pre-trained weights obtained from the previous stage. These weights are utilized to initialize the majority of layers within the segmentation model, which is subsequently fine-tuned using annotated images and unlabeled images. We incorporate a VAE branch into the segmentation model to improve the learning of feature representations from both labeled and unlabeled data. To optimize the model’s performance, we propose an adaptive learning rate strategy that dynamically adjusts according to the volume of annotated training data. Additionally, we introduce a Dice focal loss to effectively mitigate the adverse effects stemming from data category imbalances. Lastly, in the post-processing stage for segmentation result enhancement, several strategies are employed. During the testing phase, we implement a robust multi-view augmentation testing strategy to bolster the reliability of the segmentation outcomes. Furthermore, we refine these results through techniques such as maximizing connected components and filling holes in the segmentation masks. These steps are crucial for ensuring that the segmentation outputs are not only accurate but also clinically meaningful.

### 2.1. Wall-Enhanced Self-Supervised Pre-Training

Compared to model-driven methods, data-driven methods can extract more targeted features based on the task. However, achieving a superior performance with data-driven methods necessitates a larger volume of training data. This requires professional radiologists to meticulously outline the region of the bladder wall and tumor before the training of the model. Furthermore, high labeling difficulty, workload, and cost can lead to the size of the corresponding datasets being very limited, and small datasets often cannot meet the training requirements of deep neural networks. To address these issues, we propose a wall-enhanced self-supervised pre-training approach that uses contrast consistency and reconstruction observation losses. This method is designed to enhance the model’s feature extraction capabilities by means of predicting the original image, thereby improving its ability to perceive intricate details and accurately delineate the bladder boundary.

**Figure 2:**
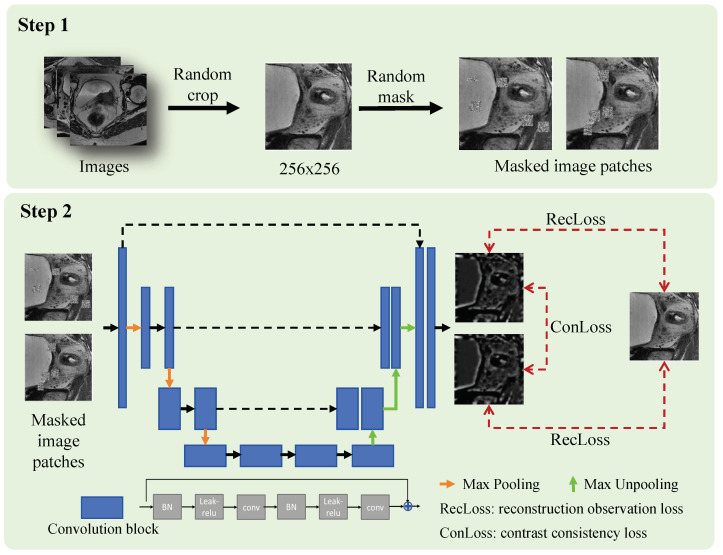
Overview of wall-enhanced self-supervised pre-training method with contrast consistency and reconstruction observation losses. Step 1: Prepare training data for image reconstruction by applying random crop and mask. Step 2: Pre-train the model via self-supervised learning constrained by contrast consistency and reconstruction observation losses.

An overview of the two-step wall-enhanced self-supervised pre-training stage is shown in Figure 2. In the first step, image processing is applied to prepare the training data for self-supervised learning. Specifically, the images are cropped randomly to image patches with a size of 256×256, then the masked image patches are obtained by adding noise at random holes. We set the number of holes to 5–10 and the size to 15–20. In the second step, these masked image patches and the cropped image patches are used as training data for self-supervised learning. Two different masked image patches obtained from the same cropped image patch are input into the reconstruction network, then the network is trained via minimization of the contrast and reconstruction loss.

The structure of the reconstruction network is borrowed from SegNet [34], which is a symmetrical network structure that can be divided at the center into a left encoder and right decoder. The input image goes through the encoder and decoder and finally through the reconstruction layer, which outputs the reconstruction result. The left encoder refers to the VGG16 for feature extraction, which is composed of nine convolution blocks and three max-pooling layers. We use max pooling to retain the main features, record the maximum index of the pooling area, and use the corresponding feature map in the subsequent upsampling process in the decoding stage. With the deepening of the network, the size of the feature map decreases gradually, and the number of channels increases gradually. The right decoder is used for image reconstruction and feature fusion, which consists of four convolution blocks and three upsampling layers. We use a max-unpooling layer to perform an upsampling operation to map the low-resolution feature map back to the resolution of the original input image. The pooling indices are used to perform the upsampling operation, inserting the maximum value of the pooled area into the corresponding position according to the index. Each convolution layer typically includes Batch Normalization (BN), an activation function, and convolution operations, with a kernel size of 3 × 3 and a filter size of 2 × 2. Furthermore, we utilize a Leaky ReLU to replace the traditional ReLU, which can be formulated as follows:

(1) $$f\left(x\right)=\left\{\begin{array}{cc} x & x\geq 0\\ ax & x<0. \end{array}\right.$$

It can solve the problem of “dead” neurons caused by negative input, ensuring that the weights are updated and enabling the network to learn more features.

#### Self-Supervised Pre-Training Loss Function

To assess the similarity between two reconstructed images, as well as between reconstructed images and their original counterparts, we present a new loss function combining contrast consistency loss and reconstruction observation loss.

In the self-supervised pre-training process, we input two image patches, $X^{1}$ and $X^{2}$, obtained from the same original image patch *X*, into the reconstruction network to obtain two reconstruction results, $\hat{X^{1}}$ and $\hat{X^{2}}$. To enhance the flexibility and generalization ability of the network, we apply the contrast consistency loss between the two reconstruction results, which is formulated as Equation (2):

(2) $$L_{con}\left(X_{1};X_{2}\right)=\sum_{i=0}^{N}\left|\hat{x_{i}^{1}}-\hat{x_{i}^{2}}\right|.$$

In addition, we use the following reconstruction observation loss to constrain the network to produce reconstruction results consistent with the original images:

(3) $$L_{rec}=L_{1}\left(X_{1};X\right)+L_{1}\left(X_{2};X\right)=\sum_{i=0}^{N}|x-\hat{x_{i}^{1}}|+\sum_{i=0}^{N}\left|x-\hat{x_{i}^{2}}\right|.$$

The total loss function of the self-supervised stage is the sum of the contrast consistency loss and reconstruction observation loss, which is shown in Equation (4):

(4) $$L_{self}=L_{con}+L_{rec}.$$

### 2.2. Semi-Supervised Multi-Region Segmentation

In the semi-supervised learning stage, the pre-trained weights from the self-supervised pre-training are transferred to the segmentation model, which will be fine-tuned using the annotated images and unlabeled images.

**Figure 3:**
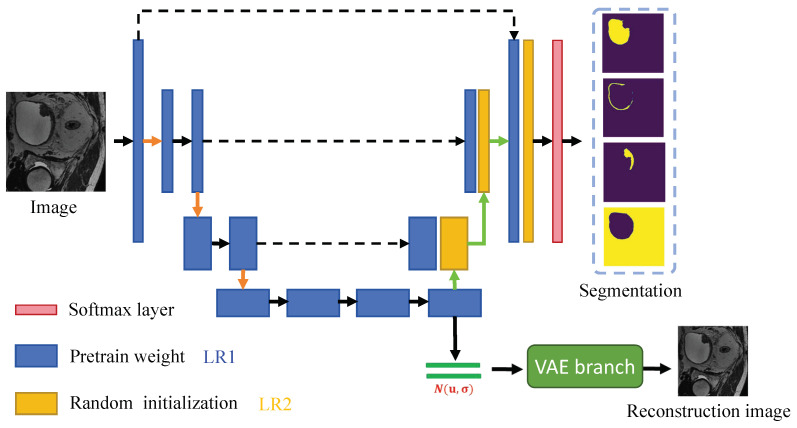
Overview of the semi-supervised multi-region segmentation stage. LR1 and LR2 are the learning rate with the pre-training weight and the random initialization weight, respectively.

**Figure 4:**
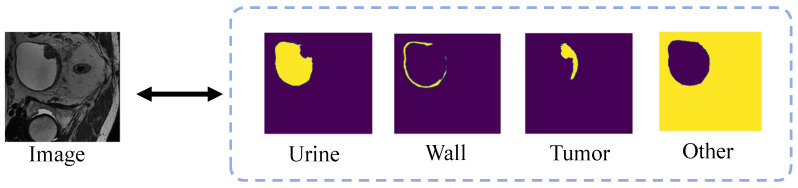
Bladder tumor and wall segmentation involves the classification of four classes: urine, bladder wall, bladder tumor, and background, with significant differences in pixel counts among each class.

An overview of the semi-supervised multi-region segmentation stage is shown in Figure 3. The architecture of the segmentation network is similar to the previous reconstruction network, with a softmax layer added at the end of the network. The blue convolution blocks are initialized with the pre-trained weights obtained from the self-supervised learning phase, while the yellow convolution blocks are initialized with random weights. To optimize the training effectiveness for annotated data, we apply different learning rates to convolution blocks with varying initialization. We add a VAE branch to enhance the feature-learning ability of the segmentation model, which is trained with annotated data and unlabeled data. To effectively segment the bladder wall and the tumor simultaneously, we divide the segmentation target into the bladder wall, tumor, urine, and background to achieve multi-region segmentation, as shown in Figure 4.

#### 2.2.1. Semi-Supervised Multi-Region Segmentation Stage Loss Function

Bladder tumor and wall segmentation involves the classification of four classes: urine, bladder wall, bladder tumor, and background, with significant differences in pixel counts among each class, as shown in Figure 4. To address this class imbalance, we apply the Dice focal loss, combining focal loss tailored for class imbalance with the commonly used Dice loss for segmentation models. Focal loss is particularly advantageous when dealing with class imbalance as it focuses on hard-to-classify samples and down-weighting the loss contribution from easily classified ones. Its formulation is given by

(5) $$FL\left(P_{t}\right)=-\alpha_{t}{\left(1-P_{t}\right)}^{\gamma }log\left(P_{t}\right),$$

where $\alpha_{urine}=0.1$, $\alpha_{wall}=0.6$, and $\alpha_{tumor}=0.8$. Dice loss is specifically designed for segmentation tasks and measures the similarity between model-generated segmentation results and real segmentation labels. Dice loss is particularly effective in capturing fine details and ensuring that the segmentation boundaries align closely with the true boundaries. It is based on the Dice coefficient, which measures the similarity of two sets. The formulation of Dice loss is given by

(6) $$DL\left(x,y\right)=-\frac{|x\cap y|+e}{|x|+|y|+e}$$

where *x* and *y* are the sets of predictions and the ground truth, respectively. Dice loss ranges from 0 to 1. When the predicted result is completely consistent with the real label, the Dice loss is 0. When the two are completely inconsistent, the Dice loss is 1. Therefore, a smaller Dice loss means that the segmentation result of the model is more similar to that of the real label, and the model performance is better. The Dice focal loss is formulated as follows:

(7) $$DFL\left(x,y\right)=FL\left(P_{t}\right)+DL\left(x,y\right).$$

By combining these two loss functions into a focal Dice loss, we aim to leverage their complementary strengths.

In addition, we also reconstruct the features of the last layer of the encoder using the VAE branch, and introduce the VAE loss to constrain the model to improve its feature-learning ability. Semi-supervised learning of the model is implemented via minimizing the VAE loss using both annotated data and unlabeled data. The formula for VAE loss is as follows:

(8) $$VAL\left(X,\hat{X}\right)=MSE\left(X,\hat{X}\right)+KL\left(N\left(\mu_{1},\sigma_{1}^{2}\right)N\left(0,1\right)\right),$$

where MSE is the mean squared error loss function, and KL stands for the Kullback–Leibler divergence regular terms.

Therefore, the total loss function of the semi-supervised multi-region segmentation stage is

(9) $$L_{semi}=DFL+VAL,$$

#### 2.2.2. Adaptive Learning Rate

As depicted in Figure 3, we apply different learning rates to convolution blocks with different initialization. We define the learning rate of the layers initialized via pre-training as LR1 and that of the layers initialized via random initialization as LR2. Furthermore, we introduce a parameter that determines the ratio between these two learning rates, which is dependent on the quantity of annotated data available:

(10) $$\beta =\frac{LR2}{LR1}=f\left(n\right),$$

where *n* is the number of annotated training data with a label and β is set to 0.02 when using all annotated data.

### 2.3. Post-Processing Stage

To further improve segmentation accuracy, we employ a series of post-processing operations to refine the segmentation results of the model. Firstly, during the testing phase, we introduce test data augmentation to improve the robustness of the segmentation. Secondly, to further correct the segmentation results, we apply post-processing techniques such as extracting the largest connected components and filling holes.

#### 2.3.1. Test Data Augmentation

**Figure 5:**
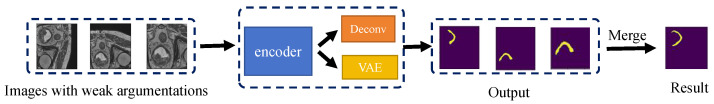
Inference process of the model with test data augmentation.

Data augmentation applies a variety of transformations to the data, enriching the variations of the input data. Figure 5 illustrates the model inference process with test data augmentation. To ensure more accurate segmentation results during testing, we first apply weak data augmentation to the test images. Subsequently, these augmented images are fed into the segmentation model to generate multiple segmentation results. We then reverse-transform these results to match the original image space and finally merge multiple outputs to derive the ultimate segmentation result.

#### 2.3.2. Segmentation Correction

**Figure 6:**
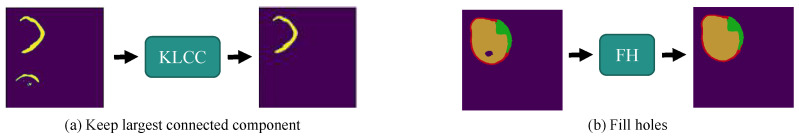
Segmentation correction processing.

Considering the inherent structural properties of bladder tumors and the bladder, given a bladder MRI image with only one bladder tissue per image, the bladder wall, tumor, and urine should all appear as neighboring regions and no holes should appear in their collection. These properties should be reflected in the segmentation results. However, these inherent features are not explicitly considered during model training; thus, we consider post-processing operations in our method. After obtaining initial segmentation results from the model, we perform post-processing operations including keeping the largest connected components and filling holes, as shown in Figure 6. These operations can refine the final segmentation results, making them more accurate and realistic.

## 3. Results

### 3.1. Dataset

This study was approved by the Ethics Committee of Tangdu Hospital of Fourth Military Medical University and involved a cohort of 60 subjects diagnosed with bladder cancer between October 2013 and May 2016. Among these subjects, there were 12 individuals with multiple cancer regions, totaling 76 identified lesions ranging from 0.57 cm to 6.05 cm in diameter. Subjects with catheter insertions and severe artifacts were excluded due to protocol misalignment. Ultimately, 52 subjects were included, with 42 allocated to the training cohort and 10 to testing for five-fold cross-validation. The cohort consisted of 19 cases of NMIBC, 30 cases of MIBC, and 3 cases with unclear pathology records. Without knowledge of the subject’s pathology, the initial areas of the urine, bladder wall, and tumor were independently and manually delineated by two radiologists with 9 years of experience using MATLAB 2016B. Then, they reached a consensus based on the corresponding pathology report and determined the final regional delineation. We invited a more experienced radiologist to proofread the annotation to further ensure accuracy. We collected 54 more subjects with NMIBC without annotation and used their images for semi-supervised learning.

All subjects underwent pre-treatment scans using a clinical whole-body MRI scanner (GE Discovery MR750 3.0 T, Boston, MA, USA). High-resolution 3D axial cube T2-weighted MRI sequences were employed for their superior soft-tissue contrast and capability in identifying muscle invasion. The duration of the 3D scans ranged from 160.456 s to 165.135 s, with repetition times and echo times set at 2500 ms and 135 ms, respectively. Each scan comprised 80 to 124 slices, each with dimensions of 512 × 512 pixels and a pixel resolution of 0.5 mm × 0.5 mm. The slice thickness and spacing between slices were standardized at 1 mm.

### 3.2. Experimental Details

We trained all models on a platform with Python 3.9 with PyTorch 1.13 and an NVIDIA GeForce RTX 4080 GPU 16 GB (Santa Clara, CA, USA) with a batch size of 16. We adopted the Adam optimizer in the self-supervised pre-training stage and set the learning rate to 0.0001 and the maximum epoch number to 100. We used the AdamW optimizer in the semi-supervised segmentation stage and set the maximum epoch number to 100 and learning rate 1 to 0.0001. β = (0.02, 0.2, 2, 10, 20) when *n* = (42, 21, 12, 4, 1). We took 20% of the training data as validation data, and the parameters were empirically determined using cross-validation on these data.

Image preprocessing involved cropping images to dimensions suitable for specific tasks: 256 × 256 for self-supervised-learning-based image reconstruction and supervised-learning-based image segmentation. This preprocessing step optimized the dataset to align with the model architecture and processing pipeline requirements.

### 3.3. Evaluation Metrics

To test the performance of the model, the Dice Similarity Coefficient (DSC) was used. The DSC mainly calculates the overlap ratio of the ground truth to the prediction considering not only the overlap of volume, but also the consistency of the segmentation results, which can intuitively reflect the accuracy of the segmentation results and is one of the most commonly used evaluation indicators in medical image segmentation tasks. The higher the overlap rate, the better the segmentation performance. It can be formulated as Equation (11):

(11) $$DSC\left(S,G\right)=\frac{2\times |S\cap G|}{\left|S|+|G\right|},$$

where *S* and *G* and are the set of predicted values and ground truth values, respectively. The numerator is the intersection between *S* and *G* and the denominator is the union of *S* and *G*. |·| indicates the number of voxels. The values of the DSC range from 0 to 1, and higher DSC values indicate more accurate segmentation.

### 3.4. Experiment Results

#### 3.4.1. Ablation Study

**Figure 7:**
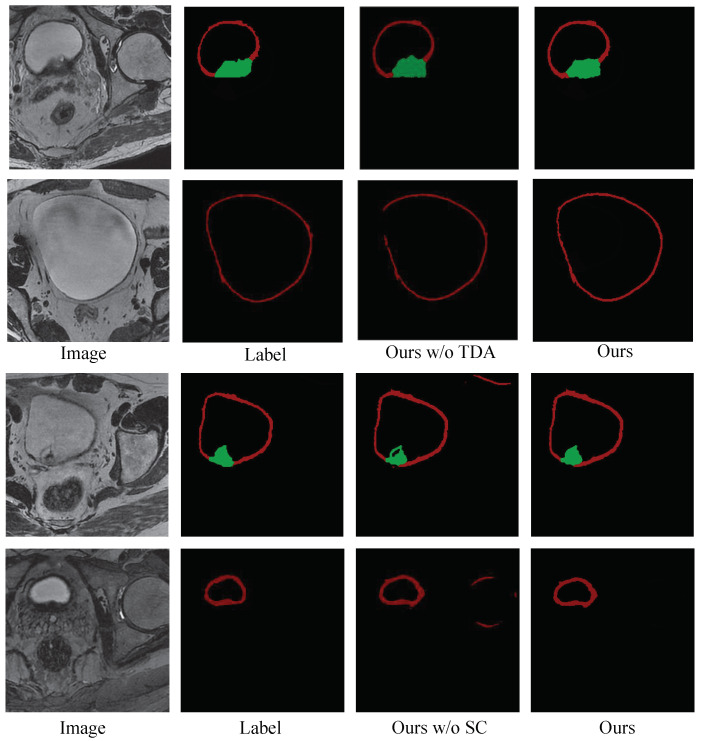
Visualization segmentation results with and without the post-processing strategy. The red area represents the bladder wall, and the green area represents the bladder tumor. TDA: test data augmentation; SC: segmentation correction.

**The Ablation Study of Post-Processing:** To verify the effectiveness of the post-processing, we conducted ablation study experiments on our model with and without post-processing techniques. The mean DSCs of our model with different settings are given in Table 1, which shows the impact of the post-processing techniques on enhancing accuracy for bladder tumor and wall segmentation. Figure 7 shows the visualization segmentation results of our model with and without post-processing. These results show that (1) using the test time augmentation strategy or segmentation correction strategy improves the performance of the model; (2) incorporating all the post-processing techniques into the model leads to a further performance gain.

**The Ablation Study of Self-supervised Pre-training:** To validate the effectiveness of this self-supervised pre-training strategy, we compared the performance of the proposed method trained with and without the self-supervised pre-training. The DSC scores calculated from the results of the bladder wall and tumor segmentation are listed in Table 2 and show that the segmentation accuracy for both the bladder wall and tumor was improved after using the self-supervised pre-training strategy.

#### 3.4.2. Comparison with Other Methods

**Figure 8:**
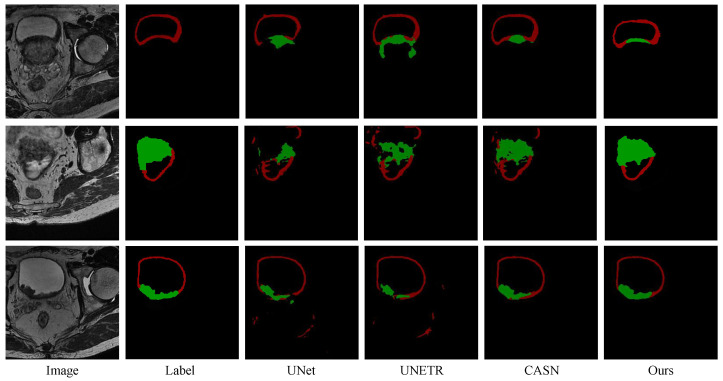
Visualization segmentation results of different methods. The red area represents the bladder wall, and the green area represents the bladder tumor.

The proposed method was compared to deep learning segmentation methods including Swin-UNet [35], Attention UNet [36], UNet [32], UNETR [37], and CSAN [33]. To ensure a fair comparison of the performance of each model, we maintained consistent settings for the training and testing data across all methods, and the results for all methods were obtained using the best model parameters determined based on the validation data. Table 2 displays the quantitative evaluation results obtained through 5-fold cross-validation with various deep learning models. Notably, our proposed method achieved the highest DSC, specificity (SPE), and sensitivity (SEN) and the lowest Hausdorff distance (HD) for both the bladder wall and the tumor. Figure 8 presents the visualization segmentation results of the different methods. As can be seen, the proposed method can obtain more accurate segmentation results than the other methods, which show better clinical rationality.

**Table 2:** Quantitative evaluation results of various deep learning models. The best results are highlighted in bold.

	Wall				Tumor			
Method	DSC	SPE	SEN	HD	DSC	SPE	SEN	HD
Swin-UNet	0.7828	0.9927	0.7947	35.8050	0.8044	0.9958	0.8143	34.4093
Attention UNet	0.7762	0.9917	0.7583	40.8166	0.7973	0.9935	0.7988	33.1058
UNet	0.7975	0.9914	0.7920	36.6742	0.8193	0.9960	0.8008	26.6833
UNETR	0.7864	0.9872	0.7663	33.1209	0.8122	0.9949	0.8196	31.7804
CASN	0.8281	0.9942	0.8018	11.4017	0.8702	0.9940	0.8526	14.4222
w/o Pre	0.8247	0.9961	0.8193	9.2195	0.8896	0.9968	0.8706	9.8994
Ours	**0.8351**	**0.9985**	**0.8499**	**7.0710**	**0.9175**	**0.9993**	**0.9485**	**5.8309**

#### 3.4.3. Experiments with Different Numbers of Annotated Data

To evaluate the performance changes of the different methods when using different quantities of annotated data, we performed experiments where different proportions of the training dataset were utilized for semi-supervised learning. All of the models were trained with varying proportions of annotated training data: 3%, 10%, 30%, 50%, and 70% of the total dataset. For each configuration, we evaluated the model’s performance on a standardized test dataset. Table 3 summarizes the DSC scores obtained by the different methods across the different annotated training data ratios.

The results reveal that leveraging self-supervised pre-training can improve the segmentation performance of the proposed model across all scenarios. Specifically, when pre-training is incorporated, even with a reduced proportion of annotated data (3% and 10%), the proposed method consistently outperforms the other methods, which rely solely on supervised learning. They also suggest that when the self-supervised learning strategy is used, the obtained DSC scores improve less and less with an increasing proportion of annotated training data. This improvement underscores the capability of self-supervised learning in learning meaningful representations from unlabeled data, which subsequently benefits supervised learning tasks via initializing the model with richer feature representations. Furthermore, qualitative analysis of the segmentation results demonstrated that the models pre-trained via self-supervision exhibit superior boundary delineation and overall segmentation quality compared to their counterparts trained exclusively with supervised data. This qualitative improvement is crucial in medical applications, where precise segmentation of anatomical structures is pivotal for accurate diagnosis and treatment planning.

#### 3.4.4. Reconstruction Results

**Figure 9:**
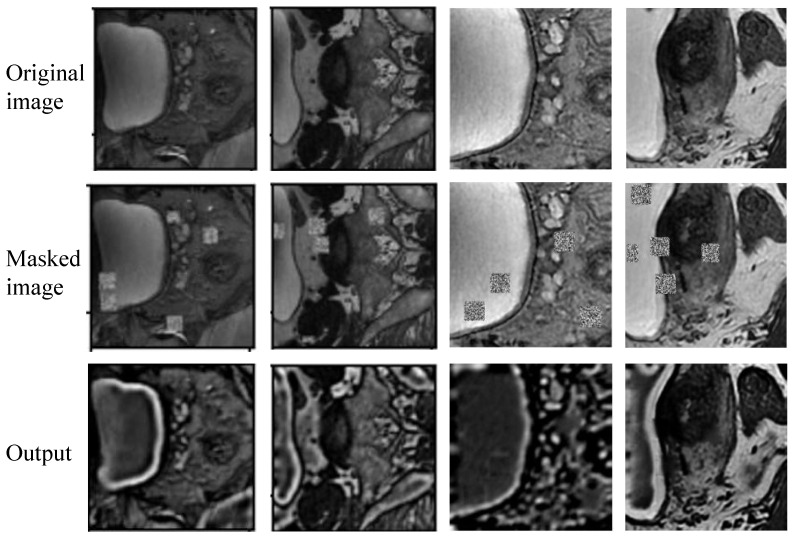
Reconstruction results in the wall-enhanced self-supervised learning stage.

Figure 9 displays the reconstruction results obtained during the wall-enhanced self-supervised learning stage. It is evident that the reconstructed images enhance the delineation of the bladder wall, increasing the contrast between the bladder wall and surrounding tissues. This enhancement indicates that the network effectively captures the boundary information of the bladder wall, thereby enhancing the capability of the subsequent bladder wall segmentation model to accurately extract boundaries.

## 4. Discussion

Bladder cancer presents a considerable challenge to public health, characterized by its high incidence and mortality rates. In clinical practice, the degree of muscle infiltration in bladder cancer is a crucial factor in determining appropriate surgical strategies and accurately staging the bladder cancer, and accurate segmentation of the bladder wall and tumor is crucial for enabling clinicians to assess whether the tumor has infiltrated muscle tissue. In this study, we introduce a semi-supervised multi-region segmentation framework for bladder wall and tumor segmentation with wall-enhanced self-supervised pre-training.

To validate the efficacy of our proposed method, we conducted a series of comparative and ablation experiments. The quantitative and qualitative results in Table 1 and Figure 7 highlight that post-processing techniques play an observable role in maintaining the structural integrity of segmentation results and the uniqueness of the bladder wall. Table 2 and Figure 8 demonstrate the accuracy and robustness of our method via implementing a five-fold cross-validation. Meanwhile, Table 3 shows that the proposed method performs well across different training data ratios when using the pre-training strategy, while Figure 9 reveals that the bladder wall is enhanced during the process of self-supervised learning. These results indicate that the proposed method achieves excellent performance in segmenting bladder walls and tumors while minimizing dependence on annotated bladder data. The improved segmentation accuracy achieved by our proposed method can lead to more reliable medical decisions, potentially improving patient outcomes and reducing the need for unnecessary interventions.

Despite these strengths, our study still has several limitations. In our current model construction, clinical prior knowledge of bladder cancer is ignored and is only considered at the post-processing stage. The lack of prior knowledge limits the overall development of the model and the effectiveness of its clinical application. In addition, there are still some data with poor annotation quality, which directly affects the learning effect and performance upper limit of the model, meaning that it is difficult for the model to achieve ideal accuracy and robustness in practical applications.

Future work will focus on integrating clinical prior knowledge more effectively into the training process to enhance the model’s ability to represent this knowledge. This could be achieved by introducing relevant clinical features and rules into the model architecture, or by adopting a hybrid model approach that combines machine learning with expert systems to better process clinical data. At the same time, it is necessary to explore and implement annotation correction algorithms to improve the effectiveness of training data and improve the performance ceiling of the model.

## 5. Conclusions

In this study, we have proposed a semi-supervised multi-region framework for bladder wall and tumor segmentation which uses wall-enhanced self-supervised learning for model pre-training. This method applies contrast consistency and reconstruction observation losses within a self-supervised learning strategy, improving the capability of the model to enhance bladder walls. Furthermore, the method incorporates semi-supervised learning with adaptive learning rate and post-processing techniques to improve the segmentation accuracy. Extensive experiments suggest that the proposed method can achieve good performance in segmenting bladder walls and tumors with minimal reliance on annotated bladder data.

## Figures and Tables

**Table 1:** An ablation study of the post-processing. This table shows the results of our model with different settings, where the best results are highlighted in bold.

Test Data Augmentation	Segmentation Correction	Wall	Tumor
		0.8292	0.8922
🗸		0.8395	0.9046
	🗸	0.8373	0.9030
🗸	🗸	**0.8351**	**0.9175**

**Table 3:** Quantitative evaluation of DSC of bladder wall and tumor with varying proportions of annotated training data. The best results are highlighted in bold.

Method	3%	10%	30%	50%	70%	90%
Wall						
UNet	0.6004	0.6464	0.7211	0.7490	0.7699	0.7881
UNETR	0.5305	0.6059	0.6291	0.7070	0.7228	0.7692
CASN	0.5600	0.6553	0.7249	0.7678	0.7974	0.8089
w/o Pre	0.5914	0.6777	0.7436	0.7797	0.8098	0.8212
Ours	**0.6532**	**0.7058**	**0.7855**	**0.8064**	**0.8177**	**0.8247**
Tumor						
UNet	0.6301	0.6477	0.7496	0.7981	0.8105	0.8156
UNETR	0.6109	0.6781	0.7051	0.7460	0.7949	0.8089
CASN	0.6651	0.6820	0.7678	0.7972	0.8264	0.8582
w/o Pre	0.6966	0.7745	0.8054	0.8378	0.8436	0.8645
Ours	**0.7343**	**0.7915**	**0.8259**	**0.8430**	**0.8526**	**0.8817**

## Data Availability

MRI data used to support the findings of this study were supplied by the Tangdu Hospital of Fourth Military Medical University under license and have not been made available because of patient privacy.

## Abbreviations

The following abbreviations are used in this manuscript:

MRI	Magnetic resonance imaging
NMIBC	Non-muscle-invasive bladder cancer
MIBC	Muscle-invasive bladder cancer
DCNN	Deep convolutional neural network
CSAN	Content and shape attention network
VAE	Variations auto-encoder
DSC	Dice Similarity Coefficient

## References

[1] Buonerba C., Ingenito C., Di Trolio R., Cappuccio F., Rubino R., Piscosquito A., Verde A., Costabile F., Iuliucci M., Crocetto F. (2024). Unraveling the Dietary Puzzle: Exploring the Influence of Diet, Nutraceuticals, and Supplements on Bladder Cancer Risk, Outcomes, and Immunotherapy Efficacy: Insights from the BLOSSOM Study and Beyond. Oncol. Ther..

[2] Bray F., Laversanne M., Sung H., Ferlay J., Siegel R.L., Soerjomataram I., Jemal A. (2024). Global cancer statistics 2022: GLOBOCAN estimates of incidence and mortality worldwide for 36 cancers in 185 countries. CA Cancer J. Clin..

[3] Flaig T.W., Spiess P.E., Agarwal N., Bangs R., Boorjian S.A., Buyyounouski M.K., Downs T.M., Efstathiou J.A., Friedlander T., Greenberg R.E. (2018). NCCN guidelines insights: Bladder cancer, version 5.2018. J. Natl. Compr. Cancer Netw..

[4] Cicione A., Simone G., Lombardo R., Franco A., Nacchia A., Ghezzo N., Zammitti F., Guidotti A., Gallo G., Molinaro E. (2023). Development of a pocket nomogram to predict cancer and disease specific survival after radical cystectomy for bladder cancer: The CRAB nomogram. Clin. Genitourin. Cancer.

[5] De Nunzio C., Franco A., Simone G., Tuderti G., Anceschi U., Brassetti A., Daneshmand S., Miranda G., Desai M.M., Gill I. (2021). Validation of the COBRA nomogram for the prediction of cancer specific survival in patients treated with radical cystectomy for bladder cancer: An international wide cohort study. Eur. J. Surg. Oncol..

[6] Mastroianni R., Brassetti A., Krajewski W., Zdrojowy R., Al Salhi Y., Anceschi U., Bove A.M., Carbone A., De Nunzio C., Fuschi A. (2021). Assessing the impact of the absence of detrusor muscle in Ta low-grade urothelial carcinoma of the bladder on recurrence-free survival. Eur. Urol. Focus.

[7] Babjuk M., Böhle A., Burger M., Capoun O., Cohen D., Compérat E.M., Hernández V., Kaasinen E., Palou J., Rouprêt M. (2017). EAU guidelines on non–muscle-invasive urothelial carcinoma of the bladder: Update 2016. Eur. Urol..

[8] Witjes J.A., Bruins H.M., Cathomas R., Compérat E.M., Cowan N.C., Gakis G., Hernández V., Espinós E.L., Lorch A., Neuzillet Y. (2021). European association of urology guidelines on muscle-invasive and metastatic bladder cancer: Summary of the 2020 guidelines. Eur. Urol..

[9] Spiess P.E., Agarwal N., Bangs R., Boorjian S.A., Buyyounouski M.K., Clark P.E., Downs T.M., Efstathiou J.A., Flaig T.W., Friedlander T. (2017). Bladder cancer, version 5.2017, NCCN clinical practice guidelines in oncology. J. Natl. Compr. Cancer Netw..

[10] Jakse G., Algaba F., Malmström P.U., Oosterlinck W. (2004). A second-look TUR in T1 transitional cell carcinoma: Why?. Eur. Urol..

[11] Fritsche H.M., Burger M., Svatek R.S., Jeldres C., Karakiewicz P.I., Novara G., Skinner E., Denzinger S., Fradet Y., Isbarn H. (2010). Characteristics and outcomes of patients with clinical T1 grade 3 urothelial carcinoma treated with radical cystectomy: Results from an international cohort. Eur. Urol..

[12] Li C., Xu C., Gui C., Fox M.D. (2010). Distance regularized level set evolution and its application to image segmentation. IEEE Trans. Image Process..

[13] Han H., Li L., Duan C., Zhang H., Zhao Y., Liang Z. (2013). A unified EM approach to bladder wall segmentation with coupled level-set constraints. Med. Image Anal..

[14] Ma Z., Jorge R.N., Mascarenhas T., Tavares J.M.R. (2011). Novel approach to segment the inner and outer boundaries of the bladder wall in T2-weighted magnetic resonance images. Ann. Biomed. Eng..

[15] Qin X., Li X., Liu Y., Lu H., Yan P. (2013). Adaptive shape prior constrained level sets for bladder MR image segmentation. IEEE J. Biomed. Health Inform..

[16] Xiao D., Zhang G., Liu Y., Yang Z., Zhang X., Li L., Jiao C., Lu H. (2016). 3D detection and extraction of bladder tumors via MR virtual cystoscopy. Int. J. Comput. Assist. Radiol. Surg..

[17] Liu Y., Zheng H., Xu X., Zhang X., Lu H. (2020). The Invasion Depth Measurement of Bladder Cancer using T2-weighted Magnetic Resonance Imaging. BioMed. Eng. OnLine.

[18] Duan C., Liang Z., Bao S., Zhu H., Wang S., Zhang G., Chen J.J., Lu H. (2010). A coupled level set framework for bladder wall segmentation with application to MR cystography. IEEE Trans. Med. Imaging.

[19] Duan C., Yuan K., Liu F., Xiao P., Lv G., Liang Z. (2012). An adaptive window-setting scheme for segmentation of bladder tumor surface via MR cystography. IEEE Trans. Inf. Technol. Biomed..

[20] Li L., Wang Z., Li X., Wei X., Adler H.L., Huang W., Rizvi S.A., Meng H., Harrington D.P., Liang Z. (2004). A new partial volume segmentation approach to extract bladder wall for computer-aided detection in virtual cystoscopy. Proceedings of the Medical Imaging 2004: Physiology, Function, and Structure from Medical Images.

[21] Long J., Shelhamer E., Darrell T. Fully convolutional networks for semantic segmentation. Proceedings of the IEEE Conference on Computer Vision and Pattern Recognition.

[22] Dolz J., Xu X., Rony J., Yuan J., Liu Y., Granger E., Desrosiers C., Zhang X., Ben Ayed I., Lu H. (2018). Multiregion segmentation of bladder cancer structures in MRI with progressive dilated convolutional networks. Med. Phys..

[23] Cha K.H., Hadjiiski L., Samala R.K., Chan H.P., Caoili E.M., Cohan R.H. (2016). Urinary bladder segmentation in CT urography using deep-learning convolutional neural network and level sets. Med. Phys..

[24] Pan H., Li Z., Cai R., Zhu Y. (2020). Accurate segmentation of bladder wall and tumor regions in MRI using stacked dilated U-Net with focal loss. Proceedings of the MIPPR 2019: Parallel Processing of Images and Optimization Techniques; and Medical Imaging.

[25] Wang Y., Ye X. (2022). MSEDTNet: Multi-Scale Encoder and Decoder with Transformer for Bladder Tumor Segmentation. Electronics.

[26] Wang Y., Li X., Ye X. (2023). LCANet: A Lightweight Context-Aware Network for Bladder Tumor Segmentation in MRI Images. Mathematics.

[27] Ronneberger O., Fischer P., Brox T. (2015). U-net: Convolutional networks for biomedical image segmentation. Medical Image Computing and Computer-Assisted Intervention–MICCAI 2015, Proceedings of the 18th International Conference, Munich, Germany, 5–9 October 2015.

[28] Hammouda K., Khalifa F., Soliman A., Abdeltawab H., Ghazal M., Abou El-Ghar M., Haddad A., Darwish H.E., Keynton R., El-Baz A. (2020). A 3D CNN with a learnable adaptive shape prior for accurate segmentation of bladder wall using MR images. Proceedings of the 2020 IEEE 17th International Symposium on Biomedical Imaging (ISBI).

[29] Hammouda K., Khalifa F., Soliman A., Ghazal M., Abou El-Ghar M., Haddad A., Elmogy M., Darwish H., Keynton R., El-Baz A. (2019). A deep learning-based approach for accurate segmentation of bladder wall using MR images. Proceedings of the 2019 IEEE International Conference on Imaging Systems and Techniques (IST).

[30] Huang X., Yue X., Xu Z., Chen Y. (2021). Integrating general and specific priors into deep convolutional neural networks for bladder tumor segmentation. Proceedings of the 2021 International Joint Conference on Neural Networks (IJCNN).

[31] Liu J., Liu L., Xu B., Hou X., Liu B., Chen X., Shen L., Qiu G. (2019). Bladder cancer multi-class segmentation in MRI with Pyramid-In-Pyramid network. Proceedings of the 2019 IEEE 16th International Symposium on Biomedical Imaging (ISBI 2019).

[32] Ma X., Hadjiiski L.M., Wei J., Chan H.P., Cha K.H., Cohan R.H., Caoili E.M., Samala R., Zhou C., Lu Y. (2019). U-Net based deep learning bladder segmentation in CT urography. Med. Phys..

[33] Dong Q., Huang D., Xu X., Li Z., Liu Y., Lu H., Liu Y. (2022). Content and shape attention network for bladder wall and cancer segmentation in MRIs. Comput. Biol. Med..

[34] Badrinarayanan V., Kendall A., Cipolla R. (2017). Segnet: A deep convolutional encoder-decoder architecture for image segmentation. IEEE Trans. Pattern Anal. Mach. Intell..

[35] Cao H., Wang Y., Chen J., Jiang D., Zhang X., Tian Q., Wang M. (2022). Swin-unet: Unet-like pure transformer for medical image segmentation. European Conference on Computer Vision.

[36] Oktay O., Schlemper J., Folgoc L.L., Lee M., Heinrich M., Misawa K., Mori K., McDonagh S., Hammerla N.Y., Kainz B. (2018). Attention U-Net: Learning where to look for the pancreas. arXiv.

[37] Hatamizadeh A., Tang Y., Nath V., Yang D., Myronenko A., Landman B., Roth H.R., Xu D. UNETR: Transformers for 3D Medical Image Segmentation. Proceedings of the IEEE/CVF Winter Conference on Applications of Computer Vision (WACV).

